# Prevalence and specificities of red cell alloantibodies in transfusion-dependent beta thalassemia patients in Yazd

**Published:** 2015-04-20

**Authors:** M Vaziri, H JavadzadehShahshahani, M Moghaddam, N Taghvaee

**Affiliations:** 1 Medical doctor of Yazd Blood Transfusion Center, High Institute for Research and Education in Transfusion Medicine, Yazd, Iran; 2 Associate professor of Blood Transfusion Research Center, High Institute for Research and Education in Transfusion Medicine, Tehran, Iran; 3 Head of reference Immunohematology laboratory of Iranian Blood Transfusion Organization, High Institute for Research and Education in Transfusion Medicine, Tehran, Iran; 4 Head of Yazd Blood transfusion center, High Institute for Research and Education in Transfusion Medicine, Yazd, Iran

**Keywords:** Exchange Transfusion, Overdose, Thalassemia

## Abstract

**Background:**

Multiple transfusions in thalassemia patients may lead to antibody production against blood group antigens and hemolytic transfusion reaction might occur. In this study, antibody screening test was performed by tube and gel methods to determine the prevalence and specificity of alloantibodies in thalassemia patients.

**Materials and Methods:**

In this cross-sectional study, overall of 100 thalassemia patients from Yazd thalassemia clinic were recruited from July to September 2013. Two blood samples with volume of 6 ml were collected from each patient for standard tube and gel method antibody screening tests and a questionnaire consisting of demographic, health and blood transfusion status was completed.

**Results:**

Out of 100 cases, 54 were female (54%) and 46 male (46%). The patients' age mean was 14.97±7.91 years with 2 to 33 years age range. Only 4% (n=4) had developed alloantibodies. (One patient developed dual alloantibody (Anti-C and Anti-D) and three patients developed single alloantibody (Anti-K)).Gel method detected 4 patients with alloantibody but in two patients not detected by the standard tube method.

**Conclusion:**

The prevalence of RBC alloantibody production in this study was less than most previous studies. Anti-K was the most prevalent alloantibody in thalassemia patients in Yazd. It seems Rh and Kell blood group phenotyping in a newly diagnosed thalassemia patient and selection of matched blood for transfusion is very important.

## Introduction

Thalassemia is the most common inherited hemoglobinopathy in the world that results from a reduced rate of one or more of the globin chains. Severe clinical manifestations of beta thalassemia major such as anemia and delayed growth are apparent in the first year of life. Lifelong red blood cell transfusion has remained the main treatment of beta major thalassemia (1, 2). Repeated blood transfusion can stimulate the patient's immune system and results in the formation of anti-erythrocyte antibodies. These alloantibodies may lead to delayed hemolytic transfusion reaction and therefore would lead to jaundice and continuous fatigue and need for more blood transfusion in these patients (2,3). Studies have demonstrated the most commonly encountered alloantibodies in the world are those directed against antigens in the Rh and Kell systems specially c, C, E, K, Kidd (Jk^a^,Jk^b^), Duffy ( Fy^a^, Fy^b^) (4-6). Blood group system antigenic difference between the donor and recipient's as well as the immune-modulatory effect of the allogenic blood transfusion on the recipient's immune system are effective factors in alloimmunization. In guidelines for chronic transfusion in patients with thalassemia, antigen phenotyping before the first blood transfusion, laboratory tests including Cell Blood Count and Red Blood Cell antibody screening test are recommended. While antibody screening is included in the compatibility testing protocol in the developed countries, it is not yet performed for all patients in Iran and most of the developing countries (6, 8, 9).

The reported frequency of antibody formation is highly variable in different parts of the world ranging from 1.13% to 40.4%. In similar studies in different parts of Iran , the prevalence of alloantibodies have been reported from 2.87%(Northeast) to 40.4%(North) (10-17). Alloantibody prevalence in other countries such as Malaysia, North America & United Kingdom, Taiwan and Egypt were 1.13%, 9.4%, 11.3-19.5%, and 16.5%, respectively (18-22). 

The purpose of this study was to determine the frequency and types of RBC alloantibodies among transfusion-dependent patients with beta-thalassemia in Yazd using two different methods.

## Materials and Methods

In this cross-sectional study, overall of 100 thalassemia patients from Yazd thalassemia clinic were recruited from July to September 2013. Two samples with volume of 6 ml were collected from each patient for standard tube and gel method antibody screening tests and a questionnaire consisting of demographic, health and blood transfusion status was completed.

The questionnaire data and test results were analyzed using SPSS (version 20) and Chi-square test was used for statistical analysis of data.

All serologic tests for total of 200 samples collected from 100 patients were performed in parallel at the immunohematology laboratory of Yazd Blood Donor Center and IBTO’s Immunohematology Reference Laboratory (IRL) of Iranian Blood Transfusion Organization (IBTO). Samples were collected exactly before blood transfusion. The average interval between latest blood transfusion and sampling was 23.7 ±11.05 days. Two 6ml blood were collected in EDTA vacutainer tubes for each patient. One sample was tested for ABO group and Rh(D) type and antibody screen test (IBTO home-made kit) with standard tube method procedure in Yazd immunohematology laboratory. The second blood sample was stored at 4°C and shipped to IRL for performance of the same tests using automated machine (BioRadHemos SPII Twinsampler,France) once a week. At IRL, the cell typing tests were performed using micro-plates with Anti-A and Anti-B [Iranian Blood Research and Fractionation Holding Company (IBRF) monoclonal, Tehran, Iran] diluted with 0.9% saline to reach already validated 30% dilution. A 0.8% RBC of A1 group cells and RBCs of B group were prepared for performance of reverse typing. At Yazd immunohematology laboratory, similar non-diluted Anti-A and Anti-B (IBRF monoclonal,Tehran,Iran) were used with 2-5% A1 cells and B cells prepared every day.

Comparing manual and automated methods no discrepancy in ABO test results of any of the patient’s samples were noticed.

Similar kits (same Lot.No) for antibody screening tests (IBTO, 3RBC cells) and antibody identification panels (IBTO, 11RBC cells) were used at both centers.

Both kits were produced by IBTO’s Immunohematology Reference Laboratory with 28 day expiration date.

The Antigen profile sheets in the kits showed at least 18 RBC antigens (9 blood group system) required by US FDA.

Parallel antibody screening tests were performed at both laboratories on duplicate samples collected from each patient. Standard tube method was performed in 3 phases. IS (Immediate Spin), 37°^C^ and Anti-Human Globulin (AHG) [Low Ionic Saline Solution(LISS); Bio-Rad] was used as enhancement media while antibody screening tests were performed by Gel method (Bio-Rad, LISS) at the IRL.

All positive antibodies screening test results were tested for antibody identification by standard tube methods in 3 phases IS, 37 °^C^ and AHG (Bio-Rad, AHG) to identify the alloantibodies. 

LISS (Bio-Rad) was used as enhancement media. Auto control tests were also included while performing antibody ID panel.

## Results

Out of 100 patients with beta-thalassemia, 54 patients (54%) were female and 46 patients (46%) were male with mean age of 14.97 ±7.9 years ranging from 2 to 33 years old (Table I).

In regard to patient's ethnicity, 52 patients (52%) were from Yazd and 48 patients (48%) were not native. Twenty four patients were from Afghanistan. ABO blood group distribution were as following: 39 patients (39%) were O type, 31 patients (31%) were B type, 21 patients (21%) were A type, and 9 patients(9%) were AB type. Ninety two percent of patients had Rh(D) antigen. 

The age of the first blood transfusion ranged from 3 to 96 months (11.25±14.15). Nine patients (9%) underwent splenectomy with no history of any alloantibody production. Two patients (2%) were found to have produced alloantibody in tube method but four patients (4%) had alloantibodies in gel method. Three of those patients were identified developing anti-K alloantibody and one patient had anti-C and anti-D alloantibodies (Table II). 

**Table I:** Clinical and laboratory characteristics of multi-transfused thalassemia patients

P-value	percent	Total	percent	Alloimmunized	percent	Not Alloimmunized		
		-		-		-	Gender	
870.0	54	54	2	2	52	52	Female	
	46	46	2	2	44	44	Male	
	-	-	-	-	-	-	Age	
781.0	13	13	0	0	13	13	≥5	
	17	17	0	0	17	17	6-10	
	27	27	1	1	26	26	11-15	
	18	18	1	1	17	17	16-20	
	14	14	2	2	12	12	21-24	
	11	11	0	0	11	11	≤25	
	-	-	-	-	-	-	Nationality	
0.923	77	77	3	3	74	74	Iranian	
	24	24	1	1	23	23	Afghan	
	-	-	-	-	-	-	Blood Group	
0.688	34	34	0	0	34	34	Rh(D) Positive	O
	5	5	1	1	4	4	Rh(D) Negative	
	20	20	1	1	19	19	Rh(D) Positive	A
	1	1	0	0	1	1	Rh(D) Negative	
	29	29	1	1	28	28	Rh(D) Positive	B
	2	2	0	0	2	2	Rh(D) Negative	
	9	9	1	1	8	8	Rh(D) Positive	AB
	0	0	0	0	0	0	Rh(D) Negative	
	-	-	-	-	-	-	Age at first blood transfusion (Year)	
0.294	75	75	3	3	72	72	<1	
	18	18	0	0	18	18	1-2	
	7	7	1	1	6	6	>2	
	-	-	-	-	-	-	Background Disease	
0.568	10	10	1	1	9	9	Hepatitis C	
	1	1	0	0	1	1	Hepatitis B	
	2	2	0	0	2	2	Heart disease	
	2	2	0	0	2	2	Diabetes	
0.521	9	9	0	0	9	9	History of Splenectomy	
	-	-	-	-	-	-	Blood Transfusion Interval(Day)	
0.260	-	23.7	-	20	-	23.7	Average Blood Transfusion Interval	

**Table II:** Clinical and laboratory characteristics of alloimmunized thalassemia patients

Antibody Screening		Background disease	Blood group &Rh	Age at first transfusion (month)	Age (year)	Nationality	Gender	Alloantibody	
Gel Method	Tube Method								
+	+	Hepatitis C	O Negative	11	20	Iranian	Female	Anti C , Anti D	1
+	-	-	B Positive	6	25	Iranian	Female	Anti K	2
+	+	-	AB Positive	6	15	Afghan	male	Anti K	3
+	-	-	A Positive	60	27	Iranian	male	Anti K	4

## Discussion

Repeated blood transfusion in thalassemia patients can cause alloimmunization against different red blood cell antigens that are absent on patients red blood cells. This alloimmunization can decrease survival of transfused red blood cells and reduces efficacy of blood transfusion. In this study, we identified five RBC alloantibodies in four multitransfused patients with beta-thalassemia. Three patients(3%) had produced a single alloantibody anti-K and one patient(1%) had produced dual antibody anti-C and anti-D. Variable rates of RBC alloimmunization in transfusion-dependent thalassemia patients have been reported, ranging from 1.13% to 40.4 % (10-22). One study on 313 thalassemia patients in the North-East of Iran reported 2.87% alloimmunization rate which all of them were against Rh blood group antigens(D,C,E) (10). Alloimmunization rate in 385 thalassemia major patients of the South-East of Iran was 17.9% which 42% of them were against Rh blood group antigens and 11.6% were against Kell blood group antigen (K) (16). In a study on 218 thalassemia major patients of the North of Iran(Sari), alloimmunization rate was 40.4% which alloantibodies against C, C^w^ and Le^a^ were the most common antibodies (17). Keikhaei et al, reported a high incidence of alloimmunization of 31-57% among 133 transfusion-dependent beta-thalassemia patients of the South-West of Iran (Ahvaz). Dominant pattern was Rh antigen alloantibodies (55%) and followed by K alloantibody (33%) (15). Alloimmunization in 711 transfusion-dependent beta-thalassemia patients of the South of Iran(Shiraz) was 5.3% which 50% and 26.3% of them were against K and Rh blood group antigens, respectively (12). Abidi et al reported that alloantibody prevalence in 90 beta-thalassemia patients of Bushehr was 9% that antibody against K antigen was most prevalent (11). Azarkeivan et al study on 441 thalassemia patients showed alloimmunization rate of 11.3% by Gel method that Anti-K and Anti-D prevalence were 28% and 16%, respectively (14). Results of three mentioned studies about predominance of alloantibody against K antigen were different from similar studies performed in Italy, Greece, Tunesia, Pakistan, and Thailand (9, 24-27). In studies of mentioned countries, alloantibodies against Rh blood group antigens were predominant. It is noticeable that anti-K was more prevalent in the South era of Iran and neighboring country, Kuwait, while in the North-East of Iran, antibodies against Rh antigens has been reported more frequently in transfusion-dependent beta-thalassemia patients. The prevalence of K antigen in the North-East of Iran is the same as European populations. Its prevalence is much higher in the South-East of Iran in comparison to other parts of Iran (13, 28). Al-joudi et al reported alloimmunization rate of 1.13%(majority of them were against Rh antigens) in 5719 admitted patients of a hospital in Malaysia.^(18) ^Alloimmunization rate in 64 chinese patients in Taiwan with thalassemia major was reported being 9.4% which all of antibodies were against Rh antigens. The presence of K antigens was the same in population, so none of patients had antibody against Kell antigens (19). Alloantibody prevalence in an Egyptian study on 501 patients with beta-thalassemia was 11.3% which Anti-K, Anti-E and Anti-C were more prevalent (21).

Thompson et al reported alloantibody prevalence of 16.5% in 697 participants with history of blood transfusion in United Kingdom and North America which majority of them were against Rh blood group system and then K antigen (20). In report of El-Danasoury, 19.5% of 235 transfusion-dependent patients with thalassemia had alloantibody that majority of them were against K and Rh system (22). In our study, one patient with history of K alloantibody in the past did not show reaction at the time of our investigation which is probably due to receiving antigen matched blood components in subsequent transfusions. HenkSchonewille et al in a 20-years study have shown that 26% of alloantibodies disappear gradually (median of 7 months) (23).

Results of the present study were similar to almost all of previous studies. They had shown majority of Red Blood Cell alloantibodies were against Kelland Rh system antigens. Although two patients (2%) of our study that had alloantibody in gel method were negative in tube method, this difference was not significant. Likewise, in Azarkeivan et al study, 52% of patients with alloantibody in gel method didn't show alloantibody in tube method (14).

 In our study, there was no significant correlation between alloimmunization and patient's age, sex, age at the first transfusion, splenectomy, background diseases, blood group, or ethnicity. 

In 75% of alloimmunized cases, the age at the first transfusion ranged from 6 to 11 months. This finding was in contrast with studies that reported blood transfusion at an early age (less than one year old) may lead to immune tolerance to repeated blood transfusions and protect them from alloimmunization (15). However, in line with Mirzaeian et al study, our results revealed no statistically significant correlation between alloantibodies and the age at the first transfusion (16).

In Azarkeivan et al study, there was no significant association among alloimmunization, splenectomy, and age of splenectomy. However in Keikhaei et al study, alloimmunization rate had significant association with history of splenectomy and beta-thalassemia intermedia (14,15). Singer et al similar to Thompson et al have been reported the significant effect of splenectomy on alloimmunization rate (6,20). In Thompson study, no significant difference was found among ethnic groups (20). In Azza Mohamed Ahmed et al research, there was significant relation between splenectomy and alloimmunization but not with number of transfused blood units (21). El Danasoury et al showed relationship between alloimmunization rate and increasing age as well as high frequency of blood transfusion and splenectomy (22).

Several factors are effective in alloimmunization: The recipient immune status, RBC antigenic difference between donor and recipient and the immunomodulatory effect of the allogenic blood transfusion on the recipient immune system (15) . Filtration is one of the most important roles of the spleen and this role is removed in splenectomized patients; this may confirm the effect of splenectomy on increasing the rate of alloimmunization (14). However, our findings did not indicate splenectomy as a risk factor for alloimmunization.

 Low alloimmunization rate in our study may be due to various reasons: 

1. Homogeneity of RBC antigens between the blood donors and recipients.

2. Regular leukoreduced blood transfusions: Reduction of leukocytes in allogenic transfusions can reduce the degree of recipient immune system activation that probably occurs through reduction of donor's antigen-presenting cells and lack of recipient's T-cell activation (6, 19, 33).

3. Probably low prevalence of K antigen in our blood donors and consequently compatible blood transfusion leads to absence or disappearance of alloantibodies even in K-negative patients. 

According to Schonewille et al study, 25% of antibodies became undetectable over the course of time (median of seven months) (23).

## Conclusion

As alloimmunization against blood groups could affect on efficacy and frequency of blood transfusion and antibodies against K & Rh systems were detected in our study, Phenotyping of a newly diagnosed thalassemia patient, specifically Kell and Rh blood group antigens and transfusion of matched blood components was very important in declining the development of RBC alloantibodies and hemolytic transfusion reactions in thalassemia patients.
